# Observation of the characteristics of the natural course of Bietti crystalline dystrophy by fundus fluorescein angiography

**DOI:** 10.1186/s12886-021-01999-z

**Published:** 2021-05-28

**Authors:** Shengjuan Zhang, Lifei Wang, Zhiqiang Liu, Huijing Sun, Qian Li, Chen Xing, Zhe Xiao, Xiaoyan Peng

**Affiliations:** 1 Beijing Institute of Ophthalmology, Beijing Ophthalmology and Visual Science Key Laboratory, Beijing Tongren Eye Center, Beijing Tongren Hospital, Capital Medical University, 17 Hougou Lane, Chongnei Street, 100005 Beijing, People’s Republic of China; 2 Hebei Provincial Key Laboratory of Ophthalmology, Hebei Provincial Eye Institute, Hebei Provincial Eye Hospital, 399 East Quanbei Street, Xingtai 054001 Hebei, People’s Republic of China

**Keywords:** Bietti crystalline dystrophy, Disease development, Fundus fluorescein angiography

## Abstract

**Background:**

Bietti crystalline dystrophy (BCD) is an autosomal recessive genetic disorder that causes progressive vision loss. Here, 12 patients were followed up for 1–5 years with fundus fluorescein angiography (FFA) to observe BCD disease progression.

**Methods:**

FFA images were collected for 12 patients with BCD who visited our clinic twice or more over a 5-year period. Peripheral venous blood was collected to identify the pathogenic gene related to the clinical phenotype.

**Results:**

We observed two types in FFA images of patients with BCD. Type 1 showed retinal pigment epithelium (RPE) atrophy in the macular area, followed by choriocapillaris atrophy and the subsequent appearance of RPE atrophy appeared at the peripheral retina. Type 2 showed RPE atrophy at the posterior pole and peripheral retina, followed by choriocapillaris atrophy around the macula and along the superior and inferior vascular arcades and the nasal side of the optic disc. The posterior and peripheral lesions of both type 1 and type 2 BCD subsequently extended to the mid-periphery; finally, all the RPEs and choriocapillaris atrophied, exposing the choroid great vessels, but type 2 macular RPE atrophy could last longer.

**Conclusions:**

The characterization of two different types of BCD development provides a better understanding of the phenotype and the progression of the disease for a precise prognosis and prediction of pathogenesis.

## Background

Bietti crystalline dystrophy (BCD) is an autosomal recessive retinal dystrophy characterized by numerous tiny sparkling yellow-white spots at the posterior pole of the fundus. The causative gene has been identified as CYP4V2 [1, 2]. In 1937, Bietti first reported three patients with BCD [3, 4]. Since then, research on the disease has continued, but the natural progression and pathogenesis of BCD remain poorly understood. Most previous work has been reported as cross-sectional studies [5–8], gene research [1, 2, 9–12], and case reports [6, 13–15]. Very few of these studies have used fundus fluorescein angiography (FFA) to characterize BCD and those that did utilized FFA images that mostly involved the posterior pole of the fundus rather than the whole retina. Few articles have reported on long-term observations of BCD [16–18], and most of those that did are only case reports and lack comparisons before and after the examinations. To our knowledge, no studies have used FFA to follow the progression of BCD in patients.

The aim of the present study was to outline the phenotype of BCD more clearly and to obtain a better understanding of the natural course of the disease. Here, we present 12 cases of BCD focusing on its progression via FFA images with the goal of identifying the features of BCD development.

## Methods

### Subjects

 The implementation of all research methods in this study followed the provisions of the Declaration of Helsinki, the Ethics Committee of Beijing Tongren Hospital, Capital Medical University, and the Ethics Committee of Hebei Provincial Eye Hospital. This study included 12 unrelated Chinese patients who were followed up at our hospital clinic over a 5-year period. The clinical characteristics and images of these patients were retrospectively analysed. For convenience, patients 1–12 were designated P1-12.

This was a retrospective analysis of 24 eyes of 12 unrelated Chinese patients who had visited our hospital clinic twice or more between January 2013 and December 2018. All patients consented for mutation screening in our laboratory. Written informed consent was obtained from each patient before peripheral venous blood was drawn for genomic DNA extraction and mutation screening of the CYP4V2 gene by direct sequencing, as previously described [19].

### Procedures

All patients underwent complete ophthalmic examinations, including best corrected visual acuity (BCVA), slit-lamp microscopy, Goldman tonometry, indirect dilatation fundus examination, and fundus photography (Kowa, Nonmyd 7, Kowa, Japan). The BCVA measurements were converted to logarithm of the minimum angle of resolution (logMAR) values [20]. The FFA images of all the patients were obtained over a 55 × 55° field with a confocal scanning laser ophthalmoscope (Heidelberg Spectralis, Heidelberg Engineering, Heidelberg, Germany). An exception was P6, whose first visit FFA was done over a 50 × 50° field with a Topcon retina camera (TRC-50DX, Topcon Corporation, Tokyo, Japan). The FFA images were used to create jigsaws each time, which were then compared to establish the developmental features of the BCD fundus lesions.

## Results

### Clinical presentations and genetic diagnoses

The general conditions and genetic diagnoses for P1–12 are shown in Tables 1 and 2. The mean patient age was 39.75 ± 12.81 years. The mean BCVA of the 24 eyes was 0.95 ± 1.06 logMAR. P5 and P7 had a family history; none of the other patients had a family history. The left eye of P6 had choroidal neovascularization (CNV) at the macular area; no CNV was found in the eyes of the other patients.

**Table 1:** Clinical data

Patient	Sex	Age	On set age	Yuzawa stage		Duration(year)	logMAR		Types
				First visit	Last visit		OD	OS	
P1	M	38	36	2	2	1	+ 1.7	+ 0.2	1
P2	F	38	37	2	2	1	+ 0.1	+ 0.5	1
P3	M	49	44	2	3	3	+ 0.2	+ 0.4	1
P4	F	45	40	2	3	5	+ 0.3	+ 0.5	1
P5	F	60	55	2	2	5	0	+ 0.1	1
P6	M	30	23	2	3	4	+ 0.8	+ 0.5	2
P7	F	31	20	3	3	4	+ 3.0	+ 3.0	Unable
P8	M	29	25	3	3	2	+ 0.4	+ 0.8	Unable
P9	F	31	25	3	3	5	+ 0.5	+ 3.0	1
P10	F	66	60	3	3	3	+ 3.0	+ 2.0	1
P11	M	26	24	3	3	1	+ 0.3	+ 0.4	2
P12	M	34	31	3	3	2	+ 0.8	+ 0.4	2

Duration: the duration of the first and last FFA examination (shown as years)

* M* male, *F* female, *logMAR* logarithm of the minimum angle of resolution, *OD* right eye, *OS* left eye, *Unable* unable to judge

**Table 2:** Genetic and consanguinity status

Patient	Genetic Analysis		Consanguinity
	Allele 1	Allele 2	
P1	c.802-8_810del17insGC	c.802-8_810del17insGC	N
P2	c.992 A > C, p. H331P	c.992 A > C, p. H331P	N
P3	c.802-8_810del17insGC	g.2979 A > G; chr4:187,115,652 A > G	N
P4	c.802-8_810del17insGC	c.992 A > C, p. H331P	N
P5	c.992 A > C, p. H331P	c.571_571delT,p. Y191Tfs*7	Y
P6	c.802-8_810del17insGC	c.802-8_810del17insGC	N
P7	c.802-8_810del17insGC	c.802-8_810del17insGC	Y
P8	c.802-8_810del17insGC	c.1091-2 A > G; g.17,344 A > G, rs199476183	N
P9	c.958 C > T, p. R320X	c.1091-2 A > G; g.17,344 A > G, rs199476183	N
P10	c.802-8_810del17insGC	c.1199G > T, R400L	N
P11	c.802-8_810del17insGC	c.802-8_810del17insGC	N
P12	c.802-8_810del17insGC	c.332T > C; p. l111T	N

*N* no, *Y* yes

The severity of the fundus appearance was graded at each patient’s first visit according to the system proposed by Yuzawa and coauthors [21] (for convenience, we call this the Yuzawa staging). Stage 1 (none of our patients): retinal pigment epithelium atrophy with white crystalline deposits is observed at the macular area. Stage 2 (P1–6): RPE atrophy extends beyond the posterior pole; choriocapillaris atrophy, in addition to the RPE atrophy, appears markedly at the posterior pole. Stage 3 (P7–12): RPE-choriocapillaris complex atrophy is observed throughout the fundus. Three patients (P3, P4, and P6) had progressed to stage 3 at the last visit (Table 1).

### Fundus colour photography

**Fig. 1:**
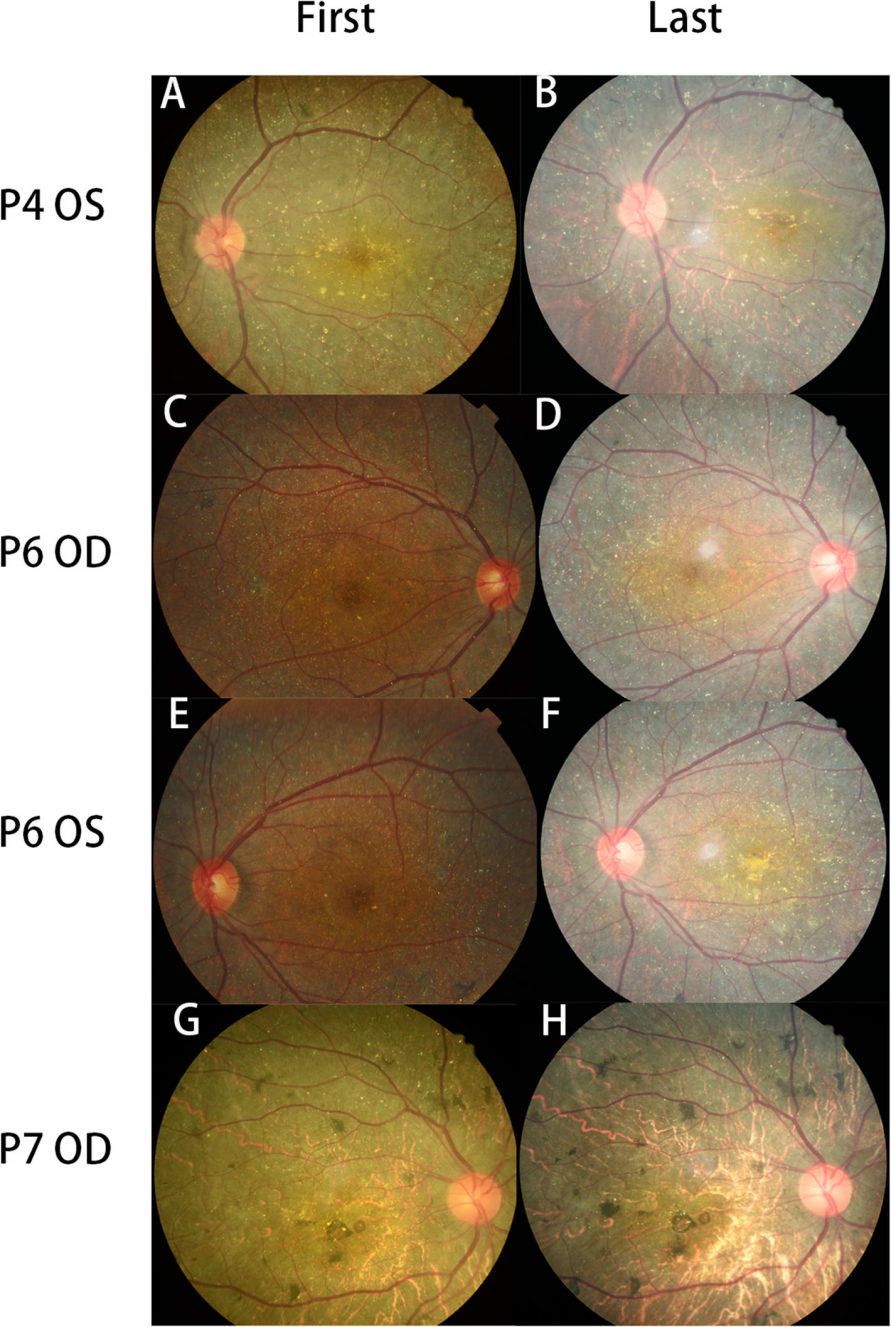
Fundus colour photographs from the first and last visits of a patient with BCD. **a, b** Choroidal great vessels appear more clearly than before. **c, d** The RPE-choriocapillaris complex atrophy of the macular area shows no significant changes, but the RPE-choriocapillaris complex of the area around the macular area is more atrophied than before. **e, f** A scar caused by choroidal neovascularization is observed at the macular area, which was not apparent at the first visit. G, H The degree of pigment clumping has increased, and the choroidal great vessels appear more clearly than at the first visit

The total number of crystalline deposits decreased over time in all eyes. At the first visit, the posterior pole retina showed a dirty bluish grey colour. At the last visit, the dirty bluish grey colour of the retina had diminished gradually, and the choroidal great vessels were clearer than before. More pigment clumps were apparent (P1-5) (Fig. 1a, b). In P6, P11, and P12, the condition of the macular area showed no significant changes, but the RPE–choriocapillaris complex of the area around it was more atrophied than before (Fig. 1c, d). A scar caused by CNV was observed the macular area in the left eye of P6 (Fig. 1 f); this was not evident at the first visit (Fig. 1e). At the last visits of P7-10, both eyes showed more pigment clumping, and the choroidal great vessels were more apparent than at the first visit (Fig. 1g, h).

### Fundus Fluorescein Angiography (FFA)

The fluorescence of both eyes in all patients was similar, except the left eye of P5 had branch retinal vein occlusion and the left eye of P6 had CNV. In this study, the FFA images at the early phases revealed hypo-fluorescence due to a filling defect of the choriocapillaris. The hypo-fluorescent lesions showed filling of the choroidal great vessels. The FFA images at the late stage indicated hyper-fluorescence staining of the hypo-fluorescent lesion margins. The mottled fluorescence in this study indicated RPE atrophy, which Mataftsi et al [5] called a “salt and pepper appearance.”

**Fig. 2:**
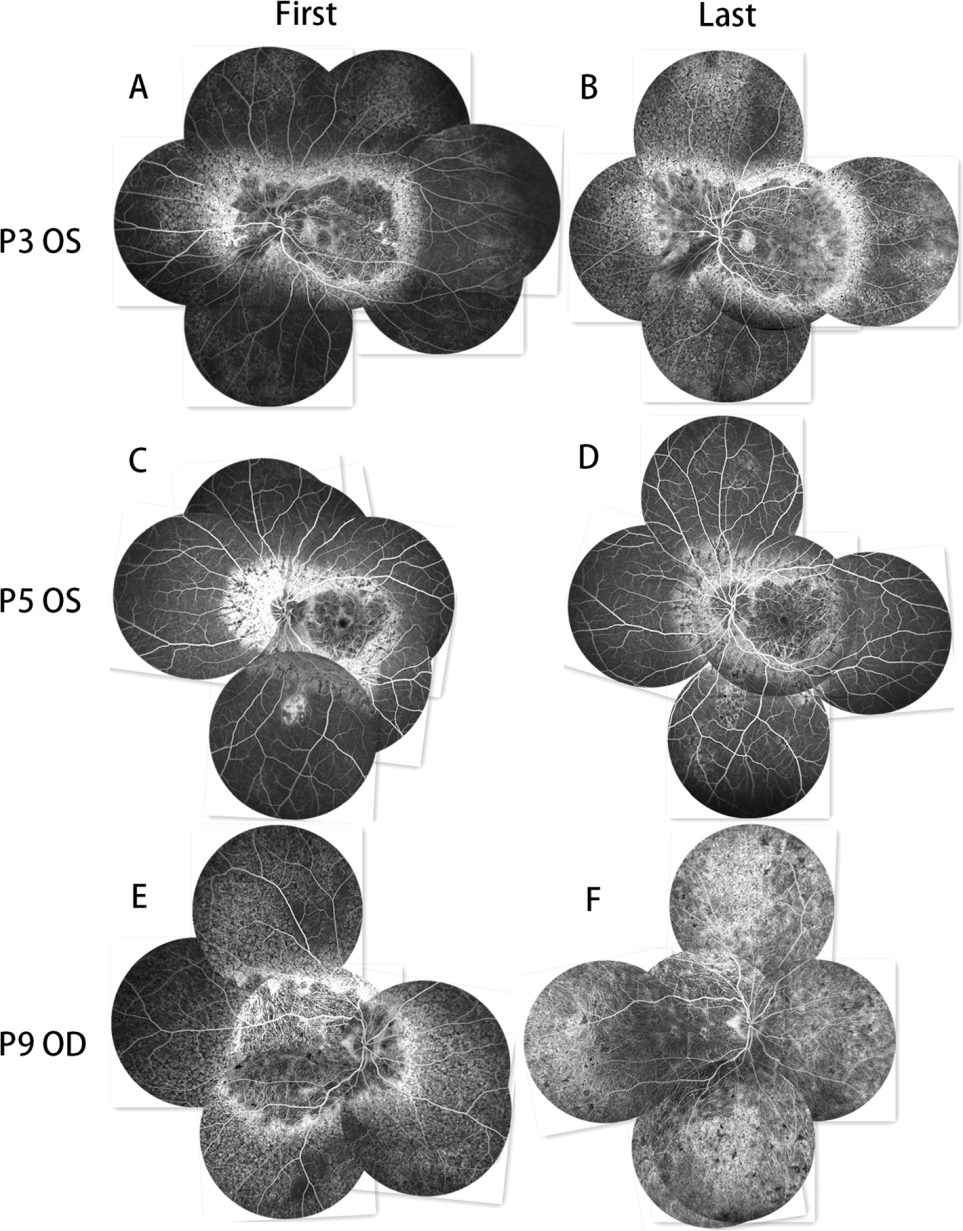
FFA image jigsaws of the first and last visits of P3, P5, and P9. **a, b** The lesion has expanded to the mid-periphery from the posterior pole and periphery. **c, d** As the posterior pole lesion develops, new lesions appear in the periphery. **e, f** The RPE-choriocapillaris complex shows significant atrophy since the first visit; the border of the choriocapillaris atrophy and RPE atrophy is not as clear as at the first visit

At their first visits, P1–P4 showed patchy hypo-fluorescence at the macular region and mottled fluorescence around it. The peripheral retina also showed mottled fluorescence, while the mid-peripheral retina showed normal retinal fluorescence (Fig. 2a, Fig. 4a). Mottled fluorescence was observed at the nasal side of P1, while P2 had mottled fluorescence and patchy hypo-fluorescence at the nasal retina and mottled fluorescence at the superior and inferior mid-periphery; however, the degree of disorder of the mottled fluorescence was slighter in the superior and inferior mid-periphery than in the posterior pole and periphery, and normal retinal fluorescence was only observed at the temporal mid-periphery. At the last visits of P1–P4, the macular and peripheral lesions had extended to the mid-periphery, and the RPE atrophy had progressed to choriocapillaris atrophy. This RPE-choriocapillaris complex atrophy was observed throughout the fundus images of P3 and P4 (Figs. 2b and 4c).

At the first visit by P5, patchy hypo-fluorescence was observed at the macular region, with mottled fluorescence around it, whereas the mid-peripheral and peripheral retinal regions showed normal fluorescence. The left eye of P5 had branched retinal vein occlusion (Fig. 2c). At the last visit of P5, the lesion of the posterior pole had extended, as had the hypo-fluorescence, and patchy mottled fluorescence was now apparent at the peripheral retina. Points of laser photocoagulation were observed at the branch retinal vein occlusion area of the left eye (Fig. 2d).

**Fig. 3:**
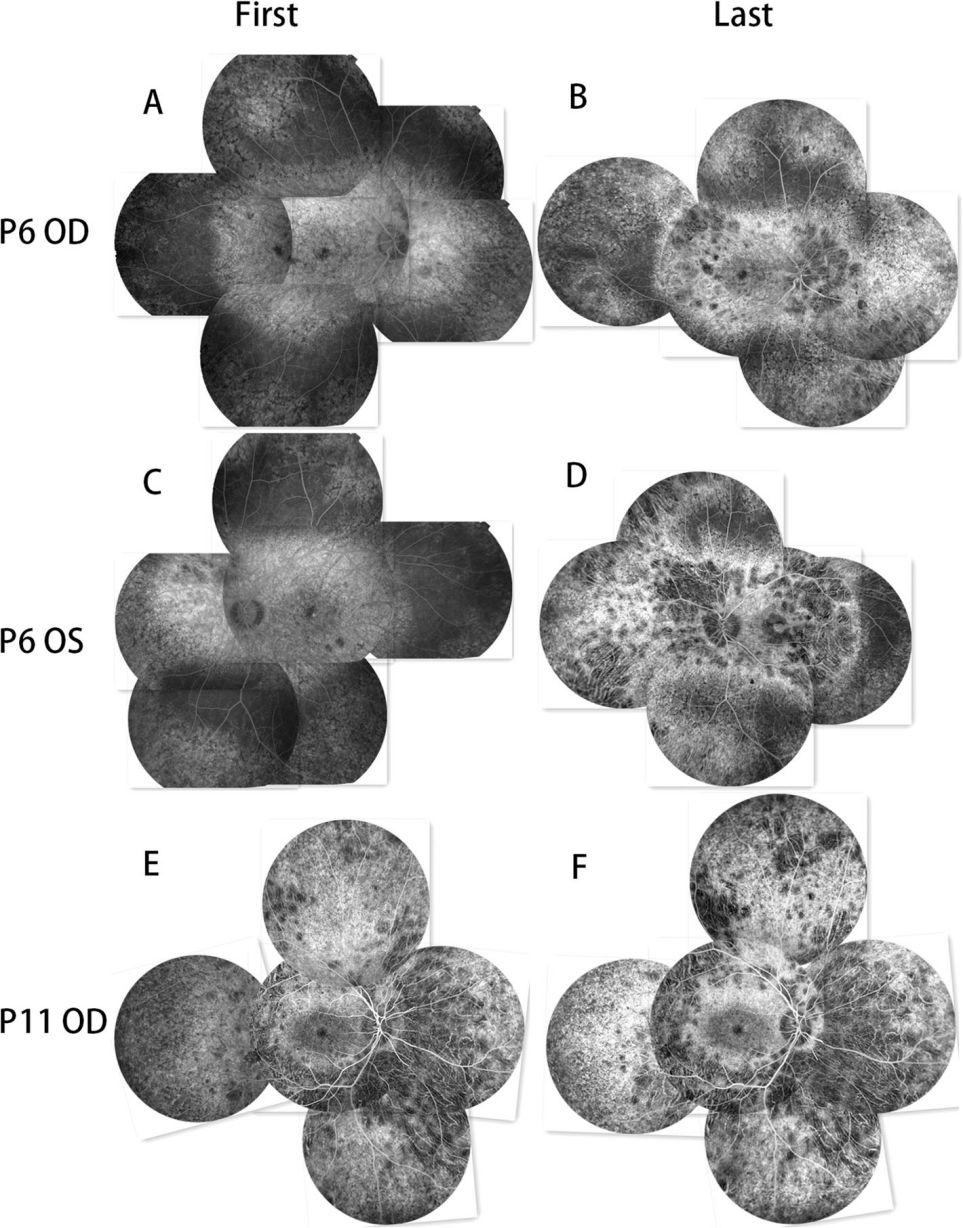
FFA image jigsaws of the first and last visits of P6 and P11. **a, c** At the first visit, both eyes of P6 show RPE atrophy at the posterior pole, the nasal side of the optic disc, and the periphery. **b, d** At the last visit, the posterior and peripheral lesions have expanded to the mid-periphery, and patches of choriocapillaris atrophy appear at the nasal side of the optic disc, the periphery, and along the superior and inferior vascular arcades. A choroidal neovascularization scar is seen at the macular area of the left eye of P6 with surrounding annular hypo-fluorescence. **e, f** The RPE atrophy of the macular area shows no significant changes, but the atrophy of the rest of the fundus area shows marked changes

At the first visit of P6, the posterior pole and the peripheral and optic disc nasal side retina showed mottled fluorescence, and a few patchy areas of hypo-fluorescence were observed at the nasal and superior nasal areas of the optic disc. The mid-periphery, except for the nasal side, showed normal retinal fluorescence (Fig. 3a, c). CNV fluorescence was observed in the macular area of the left eye (Fig. 3c), but no treatment was given. At the last visit, the posterior pole and peripheral lesion had extended to the mid-periphery, and choriocapillaris atrophy had appeared around the macular area, equivalent to the superior and inferior vascular arcade areas and the nasal area of the optic disc. At the same time, RPE atrophy progressed to choriocapillaris atrophy, but the macular RPE atrophy showed no significant changes (Fig. 3b). The CNV fluorescence of the left eye had progressed to fluorescence staining of the scar with surrounding annular hypo-fluorescence (Fig. 3d).

**Fig. 4:**
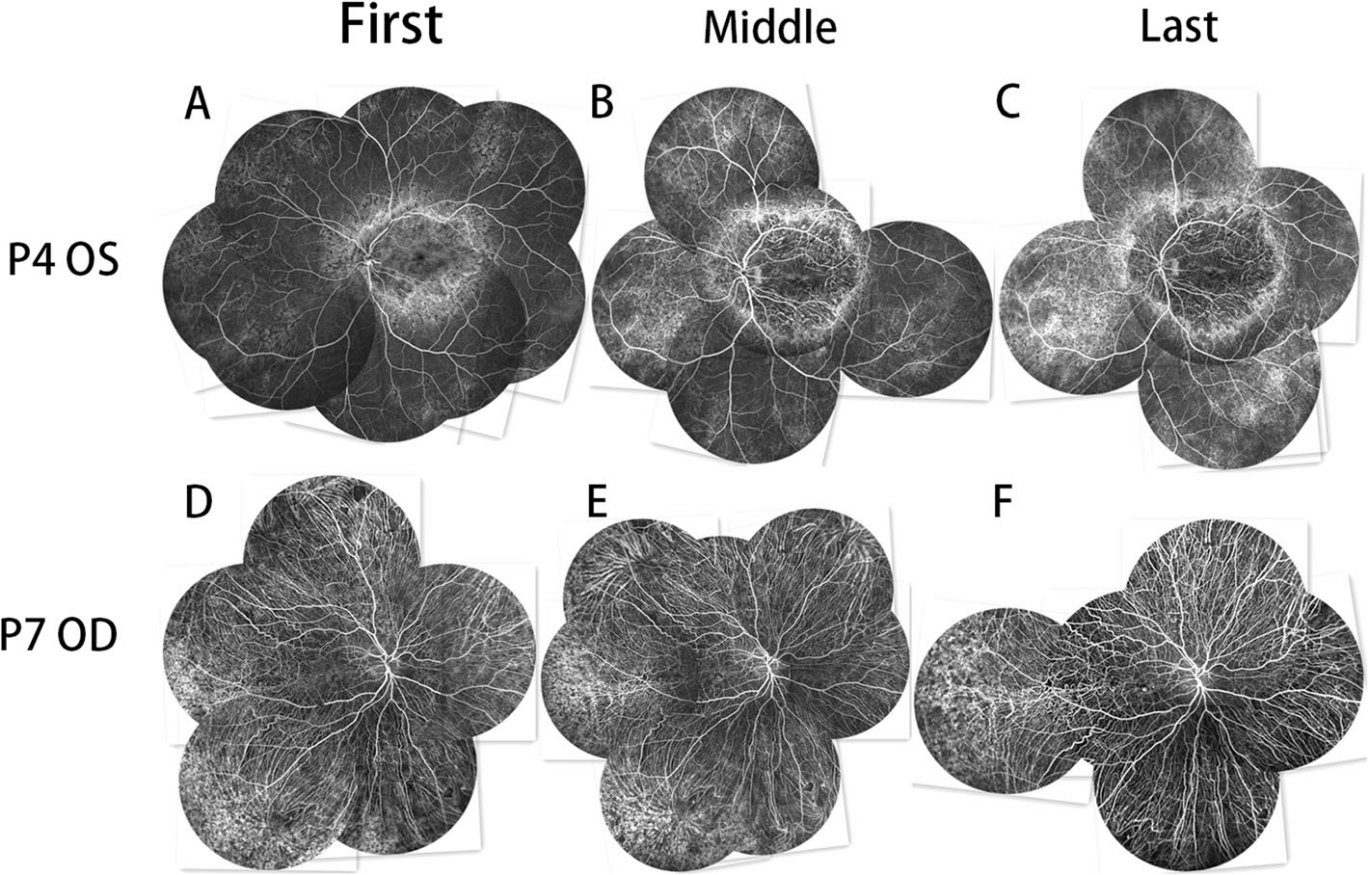
FFA image jigsaws of the first and last visits of P4 and P7. **a, b, c** Three FFA image jigsaws of the left eye of P4 taken at two-year intervals. The lesion has expanded to the mid-peripheral retina from the posterior pole retina and peripheral retina. **d, e, f** Three FFA image jigsaws of the right eye of P7 taken at two-year intervals. The majority of the choriocapillaris has atrophied. The remaining temporal peripheral choriocapillaris shows further atrophy over time

At the first visits of P7 and P8, only a few areas of mottled fluorescence remained in the peripheral retina and the superior temporal retina of the optic disc. Most of the fundus was hypo-fluorescent, and the fluorescence of the choroidal great vessels was exposed (Fig. 4d). At the last visit, the fundus hypo-fluorescence was extended, the choroidal great vessels were clearer, and the area of mottled fluorescence was reduced (Fig. 4f).

At the first visits of P9 and P10, large areas of hypo-fluorescence were seen in the posterior poles of both eyes, with the rest of the area showing mottled fluorescence (Fig. 2e). Patchy hypo-fluorescence was observed at the nasal retina in both eyes of P10. At the last visit, the hypo-fluorescence had expanded, and the area of mottled fluorescence was reduced (Fig. 2f).

At the first visits of P11 and P12, the macular region showed mottled fluorescence, with patchy hypo-fluorescence observed around the macular area (Fig. 3e). The hypo-fluorescent area was larger in P11 than in P12. At the last visit, no macular area changes were evident, and the rest of the retina showed expanded hypo-fluorescence and a more disordered mottled fluorescence (Fig. 3f).

The natural progression of P6, 11, and 12 was different from that of the others. For convenience of description, we call the natural progression mode of P1-5 and P7-10 type 1: retinal pigment epithelium atrophy at the macular area, followed by choriocapillaris atrophy, then RPE atrophy at the peripheral retina. Similarly, we call the natural progression mode of P6, 11, 12 type 2: RPE atrophy at the posterior pole and peripheral retina, followed by choriocapillaris atrophy around the macula and along the superior and inferior vascular arcades and the nasal side of the optic disc. Subsequently, the posterior and peripheral lesions of both patients with type 1 and patients with type 2 extended to the mid-periphery; finally, all the RPEs and choriocapillars atrophied, exposing the choroid great vessels, but the macular RPE atrophy of type 2 patients could persist for a longer period of time.

The disease progressed more rapidly on the nasal side of the retina of the optic disc, whereas the retina in the mid-peripheral part of the temporal side of the macular area was involved last.

## Discussion

The natural progression of BCD remains poorly understood, with few reports of follow-up appearing in the published literature. The previous staging methods for BCD have included the Yuzawa staging [21], fundus fluorescein angiography staging [5], and electrophysiological staging [19]. The Yuzawa staging is widely used [5, 19, 22–25], but most of these studies are cross-sectional studies or case reports with small numbers of cases. We followed up 12 patients with BCD to observe the natural progression of BCD.

We found that the expansion of the RPE-choriocapillaris complex atrophy was not centrifugal. This result is different from that of the previous studies. The Yuzawa staging was based on the finding of three patients [21]. Its description tends to give the impression of a centrifugal expansion. Many subsequent studies have stated that “Yuzawa described a centrifugal expansion of the RPE-choriocapillaris complex atrophy, from the macular area towards the periphery, occurring in three stages” [5, 19, 23, 26] and thus agreed with this description. We think the reason for this difference is the small numbers of patients in the previous studies, the even fewer cases of total retinal observation, and the focus on changes in the posterior pole retina in most existing studies. Halford et al [26] reported that atrophied areas of the RPE and choroid tend to develop at the posterior pole, become confluent, and expand centrifugally to involve the peripheral retina, but they only observed 55° autofluorescence in the fundus images, not a total retinal image. Consequently, the conclusion that BCD is a centrifugal expansion disease is incomplete. Mataftsi et al [5] made jigsaw observations in six patients, but they all involved advanced stages of the disease, and the entire retinal was attacked; the conclusion that BCD is a centrifugal expansion is not justified. However, Mataftsi et al [5] found one patient who showed a significant difference, as atrophic changes in the choriocapillaris were evident not only in the posterior pole but also at the equator level at the eccentricity of the vortex veins. This finding is consistent with our observation: atrophy in the posterior pole and peripheral choroid appeared before mid-peripheral atrophy.

Some reports have suggested centrifugal expansion of the visual defect based on the central scotoma seen with the 30° visual field test [1, 19, 27]. However, Liu et al [28] confirmed the visual field features using the 85° visual field test. They found that peripheral and central scotomas initially appear, but as the disease progresses, these expand and combine, ultimately resulting in visual islands only in the mid-periphery that are not found centrally. This is consistent with our observation of lesions occurring first in the centre and periphery and then eventually extending to the mid-periphery.

In the same retinal area, our FFA results showed that RPE atrophy occurs first, followed by choroidal vessel atrophy, in agreement with previous research, including that by Yuzawa and coauthors [21]. Immunohistochemistry analyses have revealed that CYP4V2 is highly expressed in the choroid and RPE, but relatively less expressed in the retinal outer and inner nuclear layers, retinal ganglion cells, and corneal epithelial cells, in accordance with the BCD phenotype [29]. The FFA images revealed changes mainly in the RPE and choroid, so RPE dysfunction has been considered the primary change in BCD [19, 26, 30]. One view holds that vascular endothelial growth factor (VEGF) is produced by the RPE and is necessary for choroidal maintenance [31]; therefore, a lack of VEGF caused by an RPE disorder may play a role in choroidal thinning.

We re-examined the FFA images in the previous literature, and we found that those images can also be divided into the two atrophy types we have mentioned before. Type 1 shows choriocapillaris atrophy first appearing at the macula [7, 11, 26, 32, 33], and type 2 shows choriocapillaris atrophy first appearing around the macular area and along the superior and inferior vascular arcades and nasal side of the optic disc [7, 14]. The numbers of patients are significantly smaller for type 2 than for type 1. For example, Wang et al [7] reported that of the 4 patients examined, 3 were type 1 and 1 was type 2. This is the same with our study. In the present study, only 3 (P6, 11, and 12) of the 12 patients were type 2. Apart from the macular area changes caused by CNV of the left eye of P6, the macular area of the other 5 eyes of the 3 patients showed slow changes, and the RPE-choriocapillaris atrophy of the mid-periphery and periphery was significantly aggravated. This suggests that type 2 patients can preserve better vision for a longer time, so these pattern differences may aid in the evaluation of the patient’s prognosis. Given the small number of patients we studied, more types can be found in the future. This needs further research in the future.

 The reason for these two different atrophy patterns is unknown. We looked at the gene mutation sites of P6, P11, and P12 and found that P6 and P11 had homozygous c.802-8_810del17insGC mutations, while P12 was heterozygous for the c.802-8_810del17insGC and c.332T > C; p. l111T mutations. In this study, P1 and P7 also had homozygous c.802-8_810del17insGC mutations, but their phenotypes differed from those of P6 and P11 (Table 2). Therefore, the specific causes of these differences need further observation and research.

In this study, we also used the Yuzawa staging as a cross-sectional staging method according to the width and depth of the BCD lesions. Since the Yuzawa staging has been used widely for many years, its application in this study was intended as a convenience for the readers to understand the condition of the eyes of our patients. It was not meant to indicate the natural progression of the disease.

In the early stage of BCD, is difficult to distinguish the type of progression in eyes with disordered pigment epithelium and no choriocapillaris atrophy. Only patients with choriocapillaris atrophy can be typed. The type of progression also cannot be determined in patients at the end stage of the disease because the choriocapillaris and RPE are atrophied and no longer visible; only the image of the choroidal great vessels is left.

This study had several limitations. First, there was a small number of included eyes and a lack of primary patient observations. However, considering the rarity of the disease and our review of the previous literature, our study on the progression of BCD using FFA picture jigsaws provides one of the largest collections of images and the largest number of patients. Second, the present study lacked multimodal imaging comparisons, but we performed cross-sectional research on multimodal patient imaging. We hope to perform multimodal imaging comparisons in the near future. Third, our study lacked patients transitioning from stage 1 to stage 2, but it is difficult to locate BCD patients with early-stage disease because the visual acuity of patients at this stage is not substantially damaged, so they seldom come to the hospital. This needs further research in the future.

## Conclusions

The natural progression of BCD in our study shows two patterns. The reasons for these different types of development need further study. However, this study provides a better understanding of the phenotype and the development of the disease. The findings presented here will be helpful for future pathogenesis research and for prognostic assessment of patients with BCD.

## Data Availability

The datasets used and/or analysed during the current study are available from the corresponding author on reasonable request.

## Abbreviations

BCD: Bietti crystalline dystrophy
FFA: Fundus fluorescein angiography
RPE: Retinal pigment epithelium
BCVA: Best corrected visual acuity
logMAR: Logarithm of the minimum angle of resolution
CNV: Choroidal neovascularization
VEGF: Vascular endothelial growth factor
